# Wound Healing: In Vitro and In Vivo Evaluation of a Bio-Functionalized Scaffold Based on Hyaluronic Acid and Platelet-Rich Plasma in Chronic Ulcers

**DOI:** 10.3390/jcm8091486

**Published:** 2019-09-18

**Authors:** Barbara De Angelis, Margarida Fernandes Lopes Morais D’Autilio, Fabrizio Orlandi, Giampiero Pepe, Simone Garcovich, Maria Giovanna Scioli, Augusto Orlandi, Valerio Cervelli, Pietro Gentile

**Affiliations:** 1 Department of Surgical Science, University of Rome Tor Vergata, Rome 00133, Italy; bdeangelisdoc@gmail.com (B.D.A.); mflmdautilio@gmail.com (M.F.L.M.D.); fabrizio.orlandi86@gmail.com (F.O.); giampieropepe.rm@gmail.com (G.P.); valeriocervelli@virgilio.it (V.C.); 2 Institute of Dermatology, F. Policlinico Gemelli IRCSS, Università Cattolica del Sacro Cuore, Rome 00168, Italy; simgarko@yahoo.it; 3 Department of Biomedicine and Prevention, Tor Vergata University of Rome, Rome 00133, Italy; scioli@med.uniroma2.it (M.G.S.); orlandi@uniroma2.it (A.O.)

**Keywords:** hyaluronic acid, platelet-rich plasma, regenerative plastic surgery, wound healing

## Abstract

Chronic ulcers are characterized by loss of substance without a normal tendency towards spontaneous healing. The Wound Bed Preparation Guideline advises that after diagnosis, the expert should correct the biological state of the ulcer micro-environment based on TIME principles (Tissue, Infection, Moisture balance, Epidermal). There are many ways to treat such ulcers, for example through use of advanced dressings, negative pressure, surgical toilets, dermal substitutes, autologous skin grafting, and free or local flaps. In vitro and in vivo pre-clinical models hold widely acknowledged potential yet complex limitations. Tissue bioengineering could be an ideal approach to foster innovative strategies in wound healing. Our observational study reports on an in vitro and in vivo evaluation of a bio-functionalized scaffold composed of platelet-rich plasma (PRP) and hyaluronic acid (HA) used in 182 patients affected by chronic ulcers (diabetic and vascular), comparing the results with a control group of 182 patients treated with traditional dressings (HA alone). After 30 days the patients who had undergone the combined treatment (PRP + HA), showed 96.8% ± 1.5% re-epithelialization, as compared to 78.4% ± 4.4% in the control group (HA only). Within 80 days, they had 98.4% ± 1.3% re-epithelialization as compared to 87.8% ± 4.1% in the control group (HA only; *p* < 0.05). No local recurrence was observed during the follow-up period. PRP + HA treatment showed stronger regenerative potential in terms of epidermal proliferation and dermal renewal compared with HA alone.

## 1. Introduction

Chronic ulcers are characterized by a loss of substance without a normal tendency towards spontaneous healing. Usually, these lesions have a vascular etiology, with 70–80% being a result of venous pathology, 15–25% due to arterial pathology, and 5–15% resulting from a mixed etiopathogenesis. All other reasons (neuropathy, infections, dysmetabolism, hematopathology and trauma) represent 5% of the total cases. A correct approach is fundamental [1]. The Wound Bed Preparation guideline advises that after diagnosis the expert should correct the biological state of the ulcer micro-environment [2] based on TIME principles (Tissue, Infection, Moisture balance, Epidermal) [3]. The most important issue is to change the two discriminant factors: super-infection and exudation. These factors create local damage to the initial continuous lesion, with subsequent ulceration. It is important to remember that elderly patients generally present comorbidity. The most common comorbidities for these patients are diabetes, hypertension, and dyslipidemia [4]. Generally, these are considered difficult patients who usually require long periods of advanced outpatient treatment.

Platelet-rich plasma (PRP) is standard routine for researchers and physicians. Preparation methods and reparative mechanisms are still in the discovery phase due to their various growth factors [4,5,6]. PRP is prepared from the patient’s blood. Growth factors stimulate tissue regeneration and positively influence the healing process of the lesion. They can reduce bleeding and may accelerate wound healing time and other beneficial therapeutic functions [4,5].

Hyaluronic acid (HA) is an important element in the extracellular matrix (ECM). Its physiological functions relate to its structural role within the ECM and its skill in interacting with cell surface receptors. Its viscous supplementation feature is able to reduce pain and improve tissue viscoelasticity. It is able to hydrate and modulate the cellular microenvironment, while its cell surface receptor bindings induce cell to-cell adhesions, cell-substrate adhesions, proliferations, and cell migrations. Therefore, HA, as a scaffold, not only facilitates the entry of a large number of cells to the wound site but also contributes to the orientation of the ECM [7] and fibrous component [8,9].

Combined use with HA and PRP in a bio-functionalized scaffold could supply many advantages for wound healing through faster healing compared with advanced dressing and single use of PRP as a bio-stimulator or HA as a scaffold, with a significant reduction in the costs of hospitalization and reduction of pain in the immediate postoperative period [7,10,11,12]. Functional capacity in terms of deambulation and joint mobility was completely restored in patients affected by exposed tendons of the foot and ankle [10]. In addition, the combined use of PRP and HA engaged in a bio-functionalized scaffold could be considered very useful in in vivo practice when autologous graft or allogeneic graft tissue might not be available in sufficient quantity to restore chronic ulcers [13]. The treatment we propose allows for easier and more rapid wound closure with excellent aesthetic improvement. Furthermore, the minimally invasive technique is well tolerated by patients.

There are many studies about the combined use of hyaluronic acid and PRP in chronic ulcers [8,10,12,14,15,16,17,18].

The aim of our study is to evaluate how platelet-rich plasma and hyaluronic acid help healing torpid chronic ulcers. In addition, this paper aims to perform an observational study with standard parameter statistics and comparable control groups using the same tools, methods, centrifugation phase, and quantities of blood, and similar basal levels of platelets in patients.

## 2. Methods

This observational case-series study was conducted following the principles outlined in the Declaration of Helsinki and internationally established ethical principles in clinical research [19]. A quality assessment was carried out based on the STrengthening the Reporting of Observational studies in Epidemiology (STROBE) checklist [20]. The study protocol, known as “Regenerative surgery of loss of substance”, formed part of an inter-university master’s degree project and was approved by Rectoral Degree (D.R. n. 1693/2016) on 12 July 2016. It was also approved by the Ethics in Research Committee of the School of Medicine at La Sapienza University of Rome, Italy, and the School of Medicine at Tor Vergata University of Rome, Italy, with registration number #27696. The latter is where the activities were performed. All patients received detailed oral and written information about the study, including the risks, benefits, and alternative therapies, and signed an informed consent form before any study procedures were performed.

### 2.1. Patient Population and Study Protocol

In total, 364 consecutive patients came to our Complex Operational Unit (UOC) at the Plastic and Reconstructive Surgery Unit of the Tor Vergata University of Rome for chronic ulcer treatment from 2010 to 2018. All of them underwent photographic and magnetic resonance imaging (MRI) in a pre and post-procedure examination of the soft tissue in order to evaluate the best way to heal torpid ulcers.

In addition, in the more complex cases a high-resolution CT (computerized tomography) scan with three-dimensional imaging for a better view of the anatomical structures was performed. Postoperative follow-up took place at 7 days, 2 and 4 weeks, 3, 6, and 12 months, and then annually. All patients were enrolled following a series of advanced dressings, which allowed for preparation of the wound bed. Intraoperative surgical debridement was performed.

Pre-operative data of the patients were acquired including information on age, gender, diagnosis, and comorbidities. The surgical procedure type (only HA or combined PRP + HA), post-operative hospital stay, pathological differentiation, and ulcer stage as well as morbidity were analyzed in order to compare the surgery risks and benefits in the two groups.

### 2.2. Patient Selection

Patients were divided in two groups: Group 1 (control group, 182 patients treated with an HA scaffold) and Group 2 (study group, 182 patients treated with the bio-functionalized scaffold HA + PRP).

**Figure 1:**
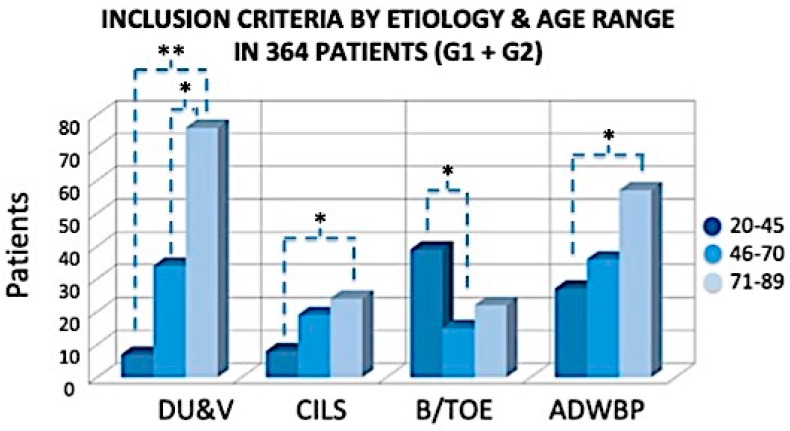
Inclusion criteria, etiology, and number of patients (total 364). DU&V: diabetic and vascular ulcers; CILS: chronic injuries with loss of substance; B/TOE: burns or ulcers with traumatic orthopedic etiology; ADWBP: advanced dressings for wound bed preparation. The exclusion criteria were divided in two categories: The first involved local factors, and the second involved systemic issues. * *p* < 0.05; ** *p* < 0.01.

Eligibility criteria (Figure 1) were identified as follows: age range between 20 and 89 years; presence of diabetic or vascular ulcer (venous ulcers, arterial-venous/mixed ulcers, and arteriolar Martorell hypertensive leg ulcers); arterial pathology with angioplasty; burns or ulcers with traumatic orthopedic etiology; and use of advanced dressings for at least two weeks for wound bed preparation (Table S1).

The authors also considered exclusion criteria. Exclusion criteria were divided in two types: local and systemic. The systemic criteria included cancer, autoimmune diseases, chemotherapy or radiotherapy, treatment with antiplatelet agents, platelet disorders, thrombocytopenia, anti-aggregating therapy, bone marrow aplasia, uncompensated diabetes, and sepsis. The local criteria included osteomyelitis, and loss of substance representing more than 50% of the segments. The authors did not considerate smokers, compensated diabetes, or genetic disorders as exclusion criteria.

### 2.3. In Vivo Application of Bio-Functionalized Scaffold PRP + HA

**Figure 2:**
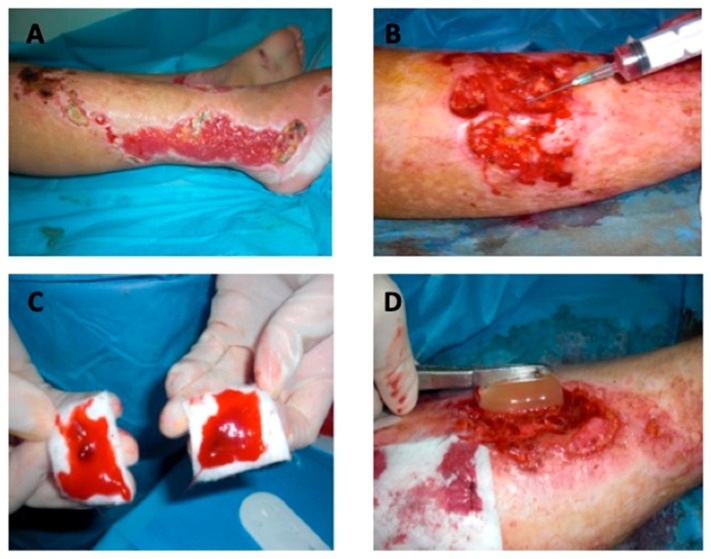
In vivo application of the bio-functionalized scaffold: (**A**) Pre-surgical view; (**B**) Intra-lesional and peri-lesional injection of platelet-rich plasma (PRP); (**C**) PRP gel applied on hyaluronic acid (HA); (**D**) Hyaluronic acid scaffold application.

The surgical technique followed the protocols of the Plastic and Reconstructive Surgery Unit at the Tor Vergata University of Rome [7,10]. The procedure started with curettage (Figure 2A) after previous disinfection and anesthesia (topical use with lidocaine chlorhydrate 5% or even sedation depending on the patient´s general condition). PRP (kit MyCells^®^) preparation involved centrifuging a patient’s blood sample (10 cc). A vial of blood was placed in a centrifuge, where it was rotated at intensely high speeds for 10 minutes at 3000 rpm. PRP as a bio-stimulator was utilized topically with intra-lesional and perilesional infiltrations in the treated area (Figure 2B) activated with calcium gluconate. For obtained PRP, gel was centrifuged (Hettich Rotofix^®^ Centrifuge) for another 15 minutes. This elastic, gelatinous, and soft cloth PRP gel was applied to the wound bed (Figure 2D). This PRP was of a very high quality, with platelet and growth factor contents equal to the highest levels obtained in previously published studies [13,14,19,20]. In particular, in all patients treated with PRP product quality checks were performed based on the following criteria:

- Evaluation of the number of platelets (in all patients the PRP had a platelet concentration equal to 1 × 10^6^/µL + 20%)
- Blood volume withdrawn (within 55 cc for each patient);
- Volume of PRP obtained (variable according to the type of use and the size of the ulcer);
- Labeling of each sample of PRP for each patient for laboratory control;
- Adverse reaction signaling (did not occur).

At the end of procedure we applied the HA scaffold (Figure 2C,D) in order to cover the side of the wound. Three-dimensional polymerized hyaluronic acid (Fidia^®^) was the biological dressing for tissue regeneration.

### 2.4. Follow-Up

HA can remain in the site for 15–20 days when growth factor action peaks; therefore, use of drug therapy can be reduced, which may be of advantage to the patient. Elastic compression was applied in order to avoid venous-lymphatic interference with the regeneration process. During the treatment, antibiotic therapy was administered in accordance with the results of culture through antibiograms, and continued for 7 days. For all patients, we followed the following steps: (1) After 7 days the secondary dressing (gauze) was changed; (2) For both groups, the silicone film (part of the hyaluronic acid matrix) was removed; (3) After 5 weeks, for Group 1 (HA only) the excess of HA was removed and replaced as necessary; (4) After 30 days, healing or apposition of new HA was applied.

### 2.5. Timing Control

The following rating system was used in order to evaluate wounds condition at 15, 30, 60, and 90 days after the treatment. We assigned a number grade based on lesion depth, with 0 = closed wound, 1 = superficial cutaneous lesion interesting epidermis, 2 = deep skin lesion interesting epidermis and derma, 3 = exposed bone and /or tendon (depending of the site of the sore), and 4 = exposure bone and/or tendon with infection.

### 2.6. In Vitro Procedures

Incisional punch biopsies of ulcers (3 mm in diameter) were obtained at baseline (pre-treatment) and post-treatment (4 weeks). Microscopic evaluation of routinely haematoxylin & eosin-stained paraffin sections [15,21] was performed to verify the healing process and images acquired by using a digital camera (E600 Eclipse, Nikon, Tokyo, Japan).

### 2.7. Statistical Analysis

The categorical data are presented as frequencies and percentages, recurrence rates after the PRP procedure with those after HA using Kaplan–Meier survival analysis, and Student’s *t*-test to investigate the healing rate of wound healing in terms of re-epithelialization over the time. Differences were considered significant if *p*-values were < 0.05.

## 3. Results

### 3.1. Wound Features

**Figure 3:**
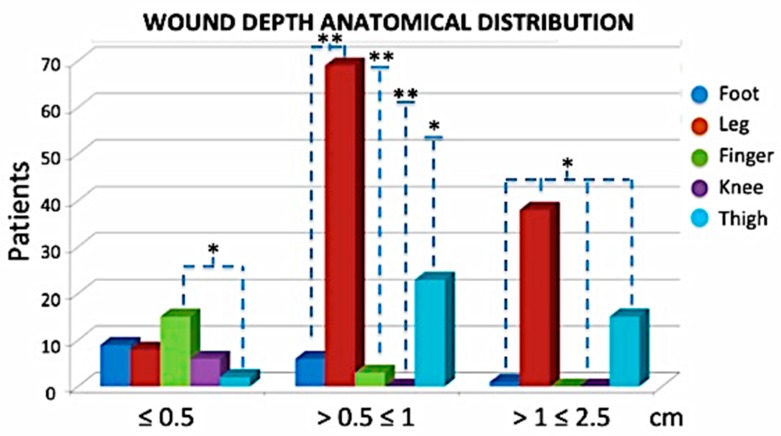
Wound depth anatomical distribution. Most of the cases were leg ulcers measuring between 0.5 and 2.5 cm in depth. * *p* < 0.05; ** *p* < 0.01.

**Figure 4:**
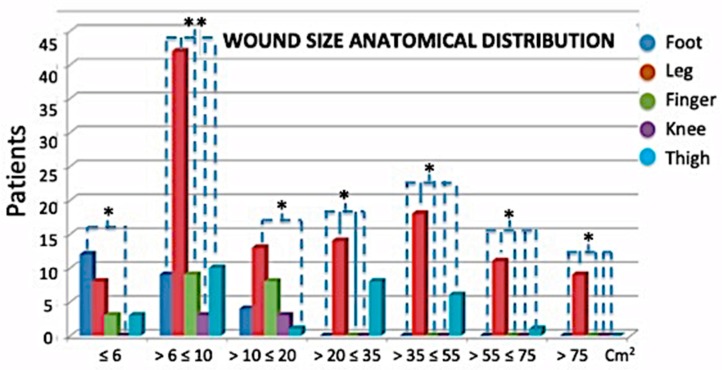
Anatomical sites (and sizes) of distribution Groups 1 and 2. The most frequent cases in both groups were chronic ulcers in the legs. Anatomical wound size distribution: 13.33% measured ≤ 6 cm^2^; 37.44% measured > 6 ≤ 10 cm^2^; 14.87% measured > 10 ≤ 20 cm^2^; 11.28% measured > 20 ≤ 35 cm^2^; 12.30% measured > 35 ≤ 55 cm^2^; 6.15% measured > 55 ≤ 75 cm^2^ and 4.62% measured > 75 cm^2^. * *p* < 0.05; ** *p* < 0.01.

**Figure 5:**
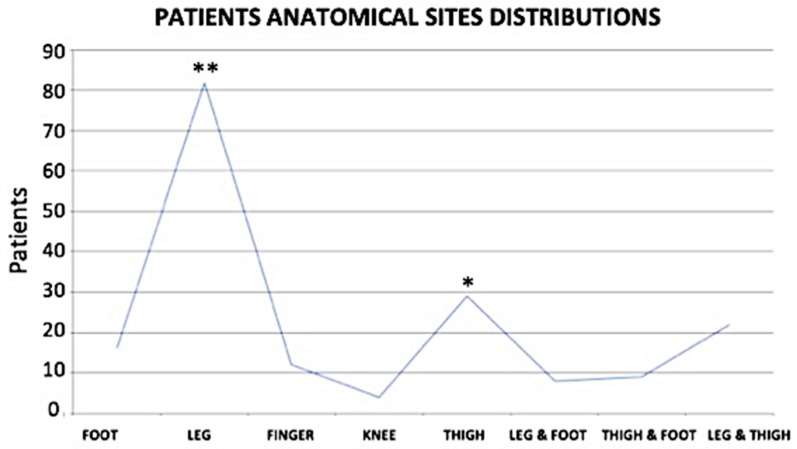
Patient Anatomical site distribution by wound size. * *p* < 0.05; ** *p* < 0.01.

In terms of wound features, the classification was based on their dimensions, the depth, the size the area, and anatomical site distribution (Figure 3, Figure 4 and Figure 5). In total, 20.51% of patients had wound depth ≤ 0.5 cm, 51.79% ≤ 0.5 ˂ 1 cm and 27.69% > 1 ≤ 2.5 cm (Figure 3).

Finally, regarding the anatomical site distribution (Figure 5), 45.05% of all patients presented with chronic ulcers on the legs; 8.79% on the feet; 6.59% on the fingers; 2.2% on the knees; 15.93% on the thighs and legs; 4.4% on the feet and ankles; 4.95% on the feet and legs; and 12.09% only on the thighs.

### 3.2. In Vivo Evaluation

Patients in the two study groups were comparable for age, indications, and ulcer grade. In total, 182 patients were enrolled and evaluated in this observational study. Overall, 52% were males and 48% were females.

**Figure 6:**
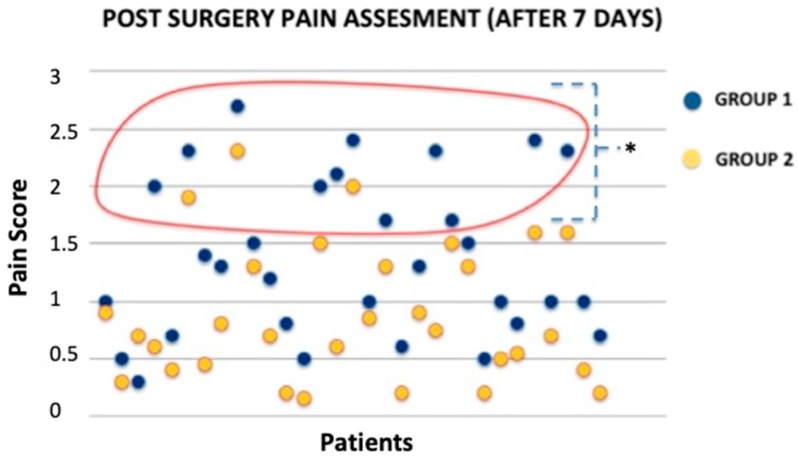
Comparison between both groups discomfort pain assessment after 7 days. Note that Group 1 patients have a higher Pain Score (PS) level. * *p* < 0.05.

To assess pain severity in patients we used a survey which was developed based on the available literature and our experience. No difference was noted for significant pain (pain score ≥ 3) during the procedure and one day after treatment. Discomfort pain (pain score ˂ 3) was more frequent in Group 1 (HA only) than in Group 2 (PRP + HA) 7 days after procedure (*p* < 0.05). Postoperative pain assessment was done in patient samples (Figure 6).

**Figure 7:**
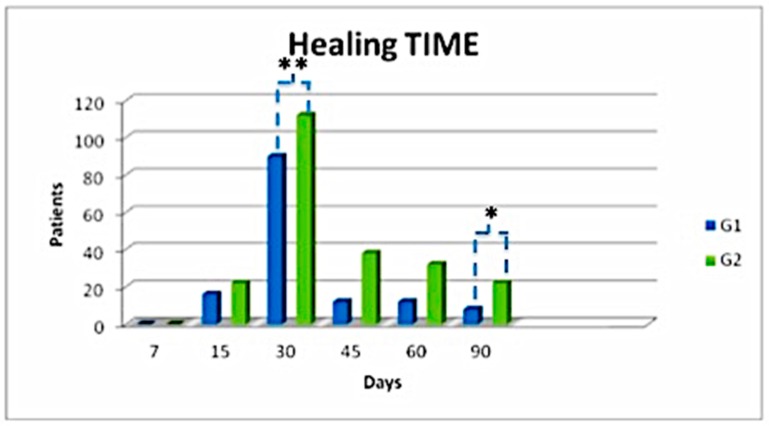
Healing time with follow-up care within 7, 15, 30, 45, 60, and 90 days. * *p* < 0.05; ** *p* < 0.01.

There were no significant complications. Only four patients had infection post-surgery (Table S1) resolved with systemic antibiotic therapy based on a preoperative antibiogram. However, some differences were noted among Group 1 (HA only) and 2 (PRP + HA). Wound conditions were evaluated at 7, 15, 30, 45, 60, and 90 days after the treatment. Note that group 2 healed faster than Group 1 (HA only; *p* < 0.05) over the time. In about 30 days, 73.62% of patients reached complete healing depending on the wound size, wound depth, and general patient condition. Note that most patients from both groups reached complete healing over 30 days. Patients who underwent combined treatment healed in a shorter time (Figure 7).

**Figure 8:**
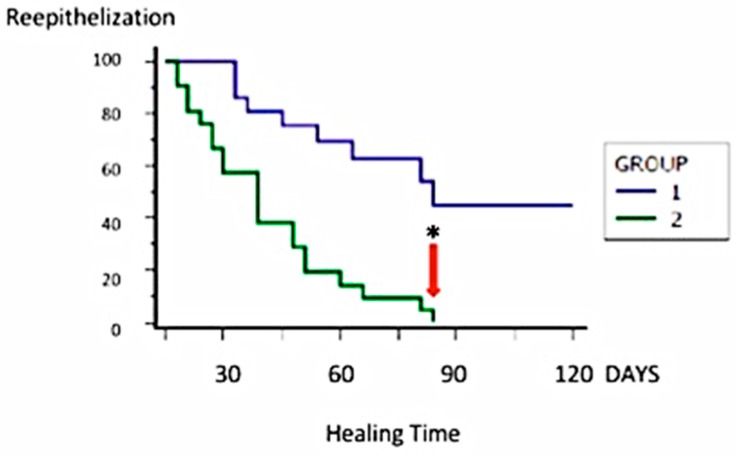
Kaplan–Meier curve depicting the likelihood of patients who have achieved a *restitutio ad integrum*. Note that the value 0 corresponds to 100% of patients with complete healing. * *p* < 0.05.

There was no difference between the healing times of different etiologies. Group 2 (PRP + HA) patients healed better than other groups (Figure 8). In addition, the youngest patients, aged between 20 and 45 years, showed faster results in term of re-epithelialization.

Overall, in Group 1 (HA only) the healing process in the first 90 days was quicker, but after that the therapy with HA alone was not sufficient. Patients from this group, in fact, needed a second surgical procedure. The arrow in Figure 8 indicates that Group 2 (PRP + HA) reached complete or partially complete healing (depending on the plague size) within 90 days. The HA (scaffold) + PRP (bio-stimulator) combination, as a bio-functionalized scaffold, halves healing time.

**Figure 9:**
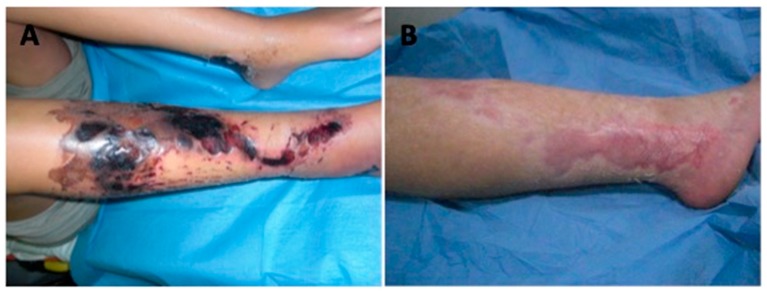
(**A**) The pre-operative situation of patient affected by full thickness and deep burn necrosis after a traumatic road crash. (**B**) Post-operative situation showing healing after 30 days with PRP + HA applications.

After 30 days, the patients who underwent a combined treatment had 96.8% ± 1.5% of reepithelization compared to 78.4% ± 4.4% in Group 1 (HA only; *p* < 0.01). The in vivo result of a patient affected by a necrotic ulcer (Figure 9A) treated with PRP + HA is reported in Figure 9B.

Within 80 days, Group 2 patients had 98.4% ± 1.3% re-epithelization compared to 87.8% ± 4.1% in Group 1 (HA only; *p* < 0.05). Only 1.6% of Group 2 (PRP + HA) patients who presented an over 20-cm^2^ wound size and an over 1-cm wound depth reached complete healing over 45 days. The median duration to complete healing was 45 days. No local recurrence was observed during the follow-up period.

Combined treatment with HA and PRP can improve the healing process in chronic ulcers (Figure 8). Regarding the healing process, the HA treatment is slower when compared to Group 2 (PRP + HA).

HA is indeed an important component in the healing process. HA acts as a scaffold for the PRP. Therefore, it stimulates rapid tissue remodeling, improving healing and allowing early restoration and return to normal function after implantation. However, it should be applied in combination to PRP in order to catalyze the wound restorative development.

### 3.3. In Vitro Evaluation

**Figure 10:**
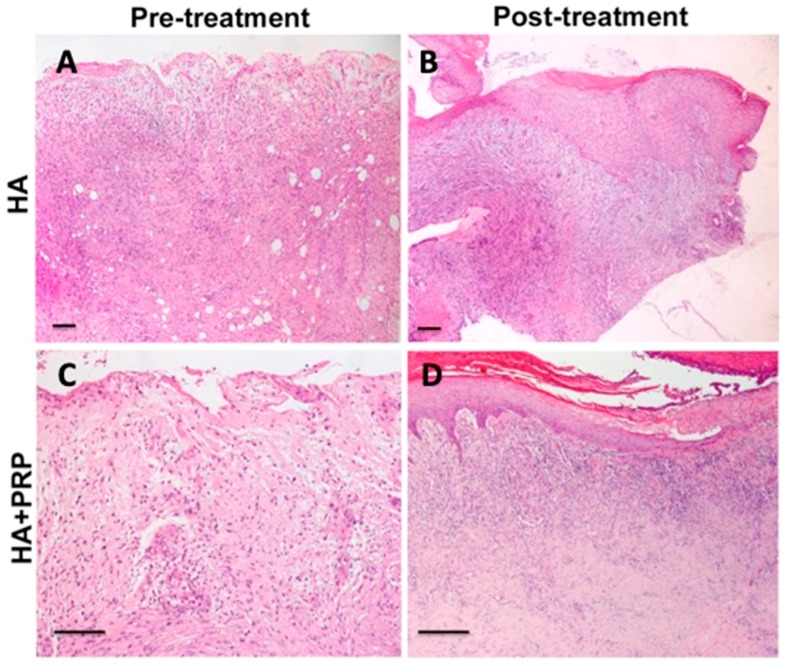
Representative microscopic images of haematoxylin & eosin-stained paraffin sections of skin biopsies at baseline. (**A**) and (**C**) Pre-treatment biopsies showed cellular debris and dermal inflammatory infiltrate; (**B**) and (**D**) Post-treatment images showed ulcer healing with re-epithelialization and new formed dermal tissue (magnification 4×). Scale bar (**A**–**D**), 100 μm.

Representative microphotographs of haematoxylin & eosin staining are reported in Figure 10. Evident healing of the ulcer was documented in post-treatment (after 3 weeks) compared with the pre-treatment skin biopsy in both the HA and PRP + HA groups. In particular, the pre-treatment biopsies showed cellular debris and dermal inflammatory infiltrate (Figure 10A,C), while in post-treatment images, regenerated skin with reactive epidermal proliferation and new formed dermal tissue were observed, along with collagen deposition and newly formed vessels (Figure 10B,D). However, PRP + HA treatment showed a stronger regenerative potential in terms of epidermal proliferation and dermal renewal compared with HA alone (Figure 10D) (*p* < 0.05).

Based on this study it can be stated that PRP combined with HA, in general, stimulates a synergistic enhancement process of tissue repair because of its similar logical and mechanism action. Furthermore, it allows a total recovery and bioavailability of the platelet growth factors, is biologically safe (not immunogenic), controls inflammation, reduces pain, and has hygroscopic, rheological, and viscoelastic properties. The combined technique acts as scaffold for cellular growth and reduces healing times, costs, and hospitalization times.

## 4. Discussion

Successful completion of this trial will provide evidence of the best treatment strategy for patients with chronic ulcers. Results from this study can lead to a better understanding of the role of HA as a scaffold and that of the combined technique with PRP as a bio-stimulator in chronic ulcer treatment [2,14]. Overall, the results demonstrated that Group 2 (PRP + HA) represents a positive system for injured cells and tissue regeneration. HA has a hydration effect [14] and stimulates properties of growth factors provided by autologous blood [10]. While hyaluronic acid acts as a temporary dermal substitute, PRP stimulates the surrounding cells. The three-dimensional scaffold is promptly colonized by fibroblasts, which generate extracellular matrix components and stimulate the systematic reconstruction of dermal tissue [22,23].

The proliferative phase of wound healing, known as the granulation phase, is marked by angiogenesis, fibroplasia, and extracellular matrix (ECM) deposition, all leading to re-epithelialization. The remodeling phase, also known as the maturation phase, is the final stage of wound healing after granulation and wound re-epithelialization or skin desquamation. During this wound repair stage, ECM is deposited and remodeled [24].

Chronic ulcers do not heal spontaneously few months [11]. Generally, these injuries are treated with advanced dressings involving hyaluronic acid, hydrocolloids, photolytic enzymes, biosynthetic or hydro fiber cellulose, expanding foam polyurethane, hydrogels, and Vac therapy [25,26,27].

Wound healing is an evolutionarily conserved complex multi-cellular process that in skin targets barrier restoration. During normal tissue repair in vivo, platelets release high concentrations of biologically active proteins including growth factors [28]. They are capable of influencing a wide range of processes and promote cell recruitment, growth, and morphogenesis [29]. The ability of platelets to release growth factors and cytokines at supra-physiological concentrations within a growing clot can be harnessed therapeutically to accelerate natural healing [30]. Of particular relevance is the epidermal growth factor (EGF) family [21,29], the transforming growth factor beta (TGF-beta) family [27,29], the fibroblast growth factor (FGF) family [27,29], vascular endothelial growth factor (VEGF) [27,29,31], granulocyte macrophage colony stimulating factor (GM-CSF) [29], platelet-derived growth factor (PDGF) [29,32], connective tissue growth factor (CTGF) [29], the interleukin (IL) family, and the tumor necrosis factor-alpha family [9,29,33]. These proteins could stimulate neo angiogenesis, and platelet-rich plasma (PRP) constitutes a three-dimensional matrix that allows cellular arrangement into a correct three-dimensional organization [30,34].

PRP is an autologous platelet concentrate contained in a small volume of plasma [6,35], enriched with growth factors and proteins that stimulate cellular processes such as chemotaxis, mitogenesis, cell differentiation, and angiogenesis [33,36,37]. For this reason, the authors identified PRP as a bio-stimulator. It is obtained through a density gradient centrifugation process of the patient’s peripheral blood, where there is a platelet concentration [29,32]. Therefore, PRP’s activation stimulates degranulation where the secretory proteins change to a bioactive state [29,32]. Active proteins are then secreted, binding to transmembrane receptors of target cells which include mesenchymal stem cells, osteoblasts, fibroblasts, endothelial cells, and epidermal cells. These agonists bind trans membrane receptors, inducing tissue repair and tissue regeneration [38,39,40].

The acquired process must be sterile and accurate. It is relatively economical, easy, and minimally invasive. Therefore, use of platelet-rich plasma is a simple and safe procedure [41]. Another mechanism that induces tissue regeneration is the acceleration of hyaluronic acid (HA) production.

HA is a biopolymer present in the extracellular matrix of skin, cartilage, bone, and brain, among other tissues [42]. HA is a wound dressing that acts as a temporary dermal substitute in wound or deep burn treatment [9]. It is a three-dimensional scaffold colonized by fibroblasts and ECM components, favoring ordered dermal tissue reconstruction [34].

During the tissue regeneration process a number of cells appear, favoring new tissue formation and reconstructing ECM assembly in order to support cellular proliferation and differentiation for the restoration induced previously. Therefore, HA facilitates entrance of a large cells number into the damaged area and supports the orientation of the ECM fibrous component [32].

Intra-articular HA injections are widely accepted for the treatment of pain associated with osteoarthritis (OA) [16]. Currently, treatment for OA is symptomatic and inadequate since it does not result in restoration of functional cartilage. In this case, the aim of HA visco-supplementation could be to reduce pain, improving the viscoelasticity of synovial fluid. PRP has been also used to treat OA to possibly induce cartilage regeneration. PRP addition is not detrimental to the visco-supplementation effect of HA [16]. A mixture of PRP and HA may be a more effective therapy than PRP or HA alone for osteoarthritis and tendinopathy [17].

HA acts as a scaffold for the PRP [32]. It is an ideal scaffold because it can accelerate remodeling, possesses increased strength, improves healing, allows renovation, and restores function after grafting [43]. HA used as a scaffold will decrease the probability of donor site morbidity, decreasing not only surgical time but also costs [22]. Combined treatment in chronic ulcers with PRP as a bio-stimulator and HA as a scaffold engaged in bio-functionalized scaffold could be considered “gold standard” in vivo practice, especially where auto graft or allograft tissue might not be available in sufficient quantity for reconstruction [20].

There are many studies suggesting regenerative medicine procedures associated with next-generation biomaterials could be used to restore an appropriate environment that encourages healing [20,21,44]. On the other hand, old and traditional studies suggest in the case of major amputation the use of a bone-anchored implant as camouflage [45,46].

The authors’ goals are to elaborate a cellular mechanism approach in order to regenerate and promote one’s own natural growth factor release. This study could demonstrate that combined treatment with PRP and HA as a bio-functionalized scaffold can be used to cover soft tissues deficits without requiring frequent dressing changes. Their combination decreases edema and chronic pain [47,48,49,50,51].

A previous study reported the use of PRP and HA for pressure ulcers [42], post-surgical complications of Achilles tendon reconstruction [12], exposed tendons of the foot and ankle [10], loss of substance with bone exposure [18], and severe hidradenitis suppurativa [52] as effective in the healing and regeneration of soft [53] and hard tissues. The healing time was shortened, and the treated area preserved a satisfying strength in plantar flexion and extension of the ankle, denoting more rapid healing with a reduction of the hospitalization costs [10,12].

This study reveals that combined treatment with PRP and HA optimizes granulation formation and tissue regeneration. The combination thus provides more rapid wound closure with an excellent aesthetic improvement. This synergetic performance allows for tissue regeneration because of their analogous action mechanisms. Both are involved in the pathophysiological mechanisms underlying wound healing [42].

Moreover, this combination helps to prevent infection, has an algiatry effect, and it is able to interact with ECM and cells forming a cell growth scaffold. This technique is minimally invasive and well tolerated by patients. In Group 2 (PRP + HA) patient granulation tissue formed faster when compared to Group 1 (HA only). Healing time was shortened: within 30 days 73.62% (± 2.1%) reached complete re-epithelization (grade 0 regarding wound condition evaluation) and the requirement for further surgical treatment was reduced depending on the wound size and depth and general patient condition. Regenerated tissue demonstrated a notable improvement in terms of texture and a shorter healing time, with good cutaneous elasticity, a significant pain decrease [50,51] immediately post-surgery, and a reduction in hospitalization costs.

In order to prevent possible complications, absorbable surgical suture use during interventions as well as collagen-based elastin and biomaterials available mainly for very deep and large wound sizes should be considered.

Our results provide evidence that PRP and HA are useful in therapeutic measures for open wounds. However, the relationship between in vitro and in vivo benefits and platelet concentration remains unclear due to inter-individual variability and the particular factors influencing each case [40].

## 5. Conclusions

This study reveals that HA has a durable effect after 6 months. Group 2 patients (treated with PRP + HA) presented an amplified effect of both components. In addition, PRP has an anti-inflammatory power and it acts much more rapidly than hyaluronic acid alone. Thus, combined treatment is painless if compared with treatment with HA alone. Treatments can be repeated only when there are no contraindications, including infection in rare cases.

This study concludes that HA acts as a scaffold for the PRP. The speedy development of granulation tissue can help to shorten the healing times and reduce the need for reconstructive surgery on supplementary soft tissue. Therefore, body repair mechanisms are stimulated to heal previously irreparable tissues. In addition, the study validates that combined treatment with PRP as a bio-stimulator and HA engaged in a bio-functionalized scaffold can reduce the healing period (*p* < 0.05) and patient pain, and is cost-effective compared to traditional treatment, increasing the patient’s quality of life.

For that reason, combined use with PRP and HA is a positive method for chronic ulcer treatment both from economic and surgical perspectives.

Additional studies to determine the ideal PRP concentration for optimal in vitro and in vivo benefits in order to standardize scientific literature would be recommended.

## Supplementary Materials

The following are available online at https://www.mdpi.com/2077-0383/8/9/1486/s1, Table S1: Database of the study group’s patients.
