# Supplementary material for: Wound Healing: In Vitro and In Vivo Evaluation of a Bio-Functionalized Scaffold Based on Hyaluronic Acid and Platelet-Rich Plasma in Chronic Ulcers
Source: J Clin Med. 2019 Sep 18;8(9):1486. doi: 10.3390/jcm8091486 (PMC6780765; doi:10.3390/jcm8091486)
Supplement: Supplementary file 1 [file jcm-08-01486-s001.pdf]

Patient	Age	Etiology	Depth (mm)	Wound size (cm2)	Anatomical site	Comorbidity	Complication
P1	80	Diabetic	1	7x4	Foot	Cardiological disease	Infection
P2	72	Vascular	0,5	10x5	Leg	Hypertension	No
P3	76	Diabetic	0,3	2x1,5	Fingers	Cardiological disease	No
P4	30	post-traumatic	2	15x4	Leg	No	No
P5	87	Vascular	0,7	7,5x4	Leg and Foot	Neurological disease	No
P6	54	Diabetic	2,3	3x2	Leg	Hypertension	No
P7	56	Vascular	1,4	9x7	Leg	Dislipidemy	No
P8	32	post-traumatic	1,3	4,5x2	Thigh and Foot	No	No
P9	75	Diabetic	2,7	5x3	Leg	Hypertension	No
P10	48	post-traumatic	1,5	6,5x4	Thigh	No	No
P11	67	Diabetic	1,2	15x8	Foot and fingers	No	No
P12	70	Diabetic	0,8	1,5x1	Leg	Cardiological disease	No
P13	36	post-traumatic	2	7x4	Thigh	No	No
P14	30	post-traumatic	2,1	13x5	Leg and Thigh	No	No
P15	80	vascular	2,4	6x3	Leg	Renal disease	No
P16	55	Diabetic	1	8x5	Foot	Hypertension	No
P17	85	post-traumatic	1,7	5x5	Thigh and Foot	Cardiological disease	No
P18	60	Vascular	0.6	13x5	Leg	No	No
P19	79	Diabetic	1,3	6x4	Foot	Renal disease	Infection
P20	43	post-traumatic	2,3	11x7	Knees	No	No
P21	39	post-traumatic	1,7	8x8	Leg	No	No
P22	76	Diabetic	1,5	9x4	Thigh	Renal disease	No
P23	62	Vascular	0,5	5x6	Leg	Cardiological disease	No
P24	54	post-traumatic	1	7x4	Leg and Knees	Respiratory disease	No
P25	83	Diabetic	0,8	8x5	Leg and Foot	Neurological disease	No
P26	78	Diabetic	2,4	3x2	Leg	No	Infection
P27	57	post-traumatic	1	6x6	Foot	Hypertension	No
P28	78	post-traumatic	2,3	9x4	Leg	Renal disease	No
P29	74	Vascular	1	15x7	Leg	Respiratory disease	No
P30	84	Diabetic	0,7	3x5	Thigh and Foot	Cardiological disease	No
P31	54	Diabetic	1,2	6x3,5	Leg	Cardiological disease	No
P32	35	post-traumatic	1,8	7x8	Leg	No	No
P33	46	Vascular	0,8	11x7	Leg	Dislipidemy	No
P34	41	post-traumatic	2	12x4	Tigh	No	No
P35	87	Vascular	1	7x10	Foot	Cardiological disease	No
P36	78	Diabetic	1,70	15x2	Leg	Cardiological disease	No
P37	68	Diabetic	1,,15	3x22	Leg	No	No
P38	25	post-traumatic	0,64	1,5x60	Thigh	Neurological disease	No
P39	49	post-traumatic	1,3	25x2	Leg and Thigh	Hypertension	No
P40	76	Vascular	2,5	15x3	Leg	Dislipidemy	No
P41	69	Vascular	1,3	12x4	Foot	No	No
P42	82	Vascular	0,7	1,5x7	Thigh and Foot	Hypertension	No
P43	87	Vascular	2,1	2,5x12	Leg	No	No
P44	73	Diabetic	1,2	4x7	Foot	No	No
P45	58	Diabetic	0,6	63x1	Knees	Cardiological disease	No
P46	72	Diabetic	1,8	48x1	Leg	Hypertension	No
P47	75	Vascular	2,5	10x5	Thigh	No	No
P48	82	Vascular	2,1	23x3	Leg	No	No
P49	69	Diabetic	1,4	14x3	Leg and Knees	Renal disease	No
P50	77	Vascular	0,9	7x4	Leg and Foot	Hypertension	No
P51	65	Vascular	2,3	10x5	Leg	Cardiological disease	No
P52	48	post-traumatic	2,4	2x1,5	Foot	No	No
P53	65	vascular	1	15x4	Leg	Renal disease	No
P54	67	Vascular	0,5	7,5x4	Leg	No	No
P55	84	Vascular	0,7	3x2	Thigh and Foot	No	No
P56	75	Vascular	1	12x9	Leg	Renal disease	No
P57	72	Vascular	1,2	4,5x2	Leg	Cardiological disease	No
P58	73	vascular	0,65	5x3	Leg	Respiratory disease	No
P59	69	vascular	2	6,5x4	Tigh	Neurological disease	No
P60	67	Diabetic	1,4	12x5	Foot	No	No
P61	64	Diabetic	2,1	1,5x1	Leg	Hypertension	No
P62	67	vascular	1,7	16x6	Fingers	Renal disease	No
P63	71	vascular	2,4	7x4	Leg	Respiratory disease	Infection
P64	66	post-traumatic	1,1	13x5	Leg and Foot	Cardiological disease	No
P65	43	vascular	1,3	6x3	Leg	Cardiological disease	No
P66	67	vascular	2,0	8x5	Leg	No	No
P67	67	vascular	0,9	5x5	Thigh and Foot	Dislipidemy	No
P68	72	Diabetic	1,6,	13x5	Leg	No	No
P69	84	Diabetic	1	6x4	Thigh	Cardiological disease	No
P70	73	Diabetic	0,5	11x7	Foot and fingers	Renal disease	No
P71	66	vascular	2,3	14x2	Leg	Hypertension	No
P72	63	post-traumatic	0,8	9x4	Leg	Cardiological disease	No
P73	71	vascular	1,45	5x6	Thigh	No	No
P74	80	vascular	1,3	7x4	Leg and Thigh	Renal disease	No
P75	65	post-traumatic	1	8x5	Leg	No	No
P76	73	vascular	0,5	3x2	Foot	No	No
P77	66	post-traumatic	0,7	6x6	Thigh and Foot	Renal disease	No
P78	74	vascular	1	9x4	Leg	Cardiological disease	No
P79	72	Diabetic	1,2	15x7	Foot	Respiratory disease	No
P80	73	Vascular	0,65	6x8	Knees	Neurological disease	No
P81	89	Diabetic	0,6	2x7	Leg	No	No
P82	65	post-traumatic	1,8	3x4	Thigh	Hypertension	No

P83	62	Vascular	2,5	12x5	Leg	Renal disease	No
P84	67	Diabetic	2,1	6x9	Leg and Knees	Respiratory disease	No
P85	74	Vascular	1,4	4x8	Leg and Foot	Cardiological disease	No
P86	78	post-traumatic	0,9	3x6	Leg	Cardiological disease	No
P87	64	Diabetic	2,5	11x3	Foot	No	No
P88	68	post-traumatic	1,7	2x12	Thigh	Dislipidemy	No
P89	84	Diabetic	0,6	14x2	Leg	No	No
P90	77	Diabetic	2	18x2	Leg	Cardiological disease	No
P91	63	Vascular	1,9	5x7	Thigh	Cardiological disease	No
P92	54	post-traumatic	0,45	16x3	Leg and Thigh	No	No
P93	48	post-traumatic	1,6	15x2	Leg	Neurological disease	No
P94	68	vascular	2,3	6x9	Foot	Hypertension	No
P95	76	Diabetic	1,3	3x8	Thigh and Foot	Dislipidemy	No
P96	68	post-traumatic	0,7	6x4	Leg	No	No
P97	78	Vascular	2,3	12x6	Foot	Hypertension	No
P98	48	Diabetic	1,4	19x3	Knees	No	No
P99	67	post-traumatic	2,1	3x21	Leg	No	No
P100	63	post-traumatic	0,6	6x12	Thigh	Cardiological disease	No
P101	78	Diabetic	2	9x4	Leg	Hypertension	No
P102	89	Vascular	0,85	5x6	Leg and Knees	No	No
P103	81	post-traumatic	1,9	7x4	Leg and Foot	No	No
P104	76	Diabetic	2,4	8x5	Leg	Renal disease	No
P105	72	Diabetic	1,6	3x2	Foot	Hypertension	No
P106	64	post-traumatic	0,75	6x6	Leg	Cardiological disease	No
P107	75	post-traumatic	1,5	9x4	Leg	No	No
P108	68	Vascular	1,8	15x7	Thigh and Foot	Renal disease	No
P109	60	Diabetic	2,4	6x8	Leg	No	No
P110	54	Diabetic	2,5	2x7	Leg	No	No
P111	33	post-traumatic	0,55	3x4	Leg and Knees	Renal disease	No
P112	74	Vascular	1,6	12x5	Leg and Foot	Cardiological disease	No
P113	81	post-traumatic	2,1	6x9	Leg	Respiratory disease	No
P114	54	Vascular	1,6	4x8	Foot	Neurological disease	No
P115	52	Diabetic	2,3	3x6	Leg	Hypertension	No
P116	63	Diabetic	1,6	11x3	Leg	Renal disease	No
P117	67	post-traumatic	1,9	2x12	Thigh and Foot	Respiratory disease	No
P118	43	post-traumatic	2,2	14x2	Leg	Cardiological disease	No
P119	70	Vascular	0,95	18x2	Leg	Cardiological disease	No
P120	75	Vascular	1,55	5x7	Leg	No	No
P121	62	Vascular	0,6	37x2	Tigh	Dislipidemy	No
P122	75	Vascular	0,8	1x45	Foot	No	No
P123	74	Diabetic	1,9	10x5	Leg	Cardiological disease	No
P124	85	Diabetic	2,6	23x3	Fingers	Renal disease	No
P125	81	post-traumatic	2,8	3x17	Leg	Hypertension	No
P126	64	vascular	1	7x4	Leg and Foot	Cardiological disease	No
P127	42	vascular	0,6	10x5	Leg	No	No
P128	67	post-traumatic	1,6	2x1,5	Leg	Renal disease	No
P129	54	vascular	0,7	15x4	Thigh and Foot	No	No
P130	81	post-traumatic	1,9	7,5x4	Leg	No	No
P131	87	vascular	0,5	3x2	Thigh	Renal disease	No
P132	67	Diabetic	1,9	15x7	Foot and fingers	Cardiological disease	No
P133	63	Vascular	2,4	6x8	Leg	Respiratory disease	No
P134	57	Diabetic	1,6	2x7	Leg	Neurological disease	No
P135	74	post-traumatic	0,75	3x4	Thigh	No	No
P136	73	Vascular	1,5	11x4	Leg and Thigh	Hypertension	No
P137	79	Diabetic	1,8	6x9	Leg	Renal disease	No
P138	80	Vascular	2,4	4x8	Foot	Respiratory disease	No
P139	49	post-traumatic	2,5	3x6	Thigh	Cardiological disease	No
P140	67	Diabetic	0,55	11x3	Leg	Cardiological disease	No
P141	54	post-traumatic	1,6	2x12	Leg and Knees	No	No
P142	77	Diabetic	2,1	14x2	Leg and Foot	Dislipidemy	No
P143	70	Diabetic	1,6	18x2	Leg	No	No
P144	63	Vascular	2,3	5x7	Foot	Cardiological disease	No
P145	85	post-traumatic	1,6	32x2	Thigh	Cardiological disease	No
P146	86	post-traumatic	1,9	1x45	Leg	No	No
P147	64	vascular	2,2	8x5	Leg	Neurological disease	No
P148	74	Diabetic	0,95	5x5	Thigh	Hypertension	No
P149	82	post-traumatic	1,55	13x5	Leg and Thigh	Dislipidemy	No
P150	66	Vascular	1,3	3x15	Leg	No	No
P151	78	Diabetic	2,5	11x7	Foot	Hypertension	No
P152	73	post-traumatic	1,3	14x2	Thigh and Foot	No	No
P153	74	post-traumatic	0,7	9x4	Leg	No	No
P154	72	Diabetic	2,1	5x6	Foot	Cardiological disease	No
P155	47	Vascular	1,2	7x4	Knees	Hypertension	No
P156	45	post-traumatic	0,6	8x5	Leg	No	No
P157	61	Diabetic	1,8	3x2	Thigh	No	No
P158	27	Diabetic	2,5	6x6	Leg	Dislipidemy	No
P159	84	post-traumatic	2,1	9x4	Leg and Knees	No	No
P160	75	post-traumatic	1,4	15x7	Leg and Foot	Cardiological disease	No
P161	49	Vascular	0,9	6x8	Leg	Renal disease	No
P162	73	Diabetic	2,3	2x7	Foot	Hypertension	No
P163	81	Diabetic	2,4	3x4	Leg	Cardiological disease	No
P164	20	post-traumatic	1	12x5	Leg	No	No
P165	78	Vascular	0,5	5x8	Thigh and Foot	Renal disease	No
P166	73	Vascular	0,7	7x3	Leg	No	No
P167	73	post-traumatic	2,3	25x2,5	Leg	No	No
P168	88	vascular	1,6	3x14	Leg	Renal disease	No
P169	25	Vascular	1,9	5x8	Leg	Cardiological disease	No

P170	64	Vascular	2,2	4x7	Leg	Respiratory disease	No
P171	33	Vascular	0,95	3x9	Tigh	Neurological disease	No
P172	74	Vascular	1,55	12x4	Foot	No	No
P173	44	vascular	1,3	8x5	Leg	Hypertension	No
P174	27	vascular	2,5	3x2	Fingers	Renal disease	No
P175	69	Diabetic	1,3	3x8	Leg	Respiratory disease	No
P176	72	Diabetic	0,7	9x4	Leg and Foot	Cardiological disease	No
P177	51	vascular	2,1	11x6	Leg	Cardiological disease	No
P178	64	vascular	1,2	6x8	Leg	No	No
P179	34	post-traumatic	0,6	2x7	Thigh and Foot	Dislipidemy	No
P180	87	vascular	1,54	3x4	Leg	No	No
P181	78	vascular	0,7	11x3	Thigh	Cardiological disease	No
P182	62	vascular	1,3	7x5,5	Foot and fingers	Cardiological disease	No
